# Impact of Mindfulness-Based Cognitive Therapy on Trajectory of Mood States in Patients with Chronic Pain: A Retrospective Observational Study of Within-Session Evaluations

**DOI:** 10.3390/jcm15051839

**Published:** 2026-02-28

**Authors:** Chisato Tanaka, Yuta Shinohara, Saki Takaoka, Morihiko Kawate, Yuki Mashima, Reiko Hoshino, Daisuke Fujisawa, Shizuko Kosugi, Kenta Wakaizumi

**Affiliations:** 1 Interdisciplinary Pain Center, Keio University Hospital, 35 Shinanomachi Shinjuku-ku, Tokyo 160-8582, Japan; c.tanaka1203@keio.jp (C.T.);; 2 Department of Anesthesiology, Keio University School of Medicine, 35 Shinanomachi Shinjuku-ku, Tokyo 160-8582, Japan; 3 Department of Neuropsychiatry, Keio University School of Medicine, 35 Shinanomachi Shinjuku-ku, Tokyo 160-8582, Japan

**Keywords:** chronic pain, effectiveness prediction, mindfulness, mood state, psychological burden

## Abstract

**Background/Objectives**: Mindfulness-Based Cognitive Therapy (MBCT) is a well-documented treatment option for chronic pain. However, few studies have examined the emotional state experienced by participants with chronic pain during the sessions. This study aimed to assess the trajectory of mood experiences in an MBCT program and evaluate the effect on temporal mood state. **Methods**: The Temporary Mood Scale (TMS) was used to assess vigor, fatigue, anger-hostility, depression-dejection, tension-anxiety, and confusion before and after each MBCT session from July 2018 to May 2019. A total of 33 patients with chronic pain who attended the MBCT program were included in the study. A mixed-effect model was used to identify single-session and overall effects on each mood state. Pearson’s correlation analysis was used to examine the associations between changes in the six mood states and baseline conditions, including pain-related factors. **Results**: The mixed-effect model identified significant improvements in single-session effects on all mood states (*p* < 0.001). Significant overall effects were identified on vigor (*p* < 0.01), depression-dejection (*p* < 0.01), tension-anxiety (*p* < 0.001), and confusion (*p* < 0.05) states throughout the sessions. Moreover, participants with higher tension-anxiety at baseline showed significantly greater improvements in anger-hostility (*p* < 0.05), depression-dejection (*p* < 0.01), tension-anxiety (*p* < 0.05), and confusion (*p* < 0.01). **Conclusions**: Individual sessions and the overall MBCT program significantly improved the mood states of patients with chronic pain. Patients with higher tension-anxiety are likely to be effective treatment targets for MBCT programs.

## 1. Introduction

Chronic pain is defined as pain that persists or recurs for more than 3 months [1]. Owing to its high prevalence, which affects around 20–30% of adults worldwide and is particularly common in middle-aged and older individuals [2,3,4], the impact of chronic pain on society in terms of quality of life and productivity is significant [5,6]. Expanding the treatment options for chronic pain is crucial for reducing pain-related disability [7], maintaining individuals’ quality of life and work productivity [6,8], and alleviating the long-term social and economic burden associated with chronic pain [9,10]. In particular, the importance of psychological therapies for chronic pain has been increasingly recognized [11,12]. Patients with chronic pain commonly suffer persistent negative mood states, such as anxiety, depression and anger, which are closely linked to greater pain-related disability and poorer quality of life [13,14]. Given that emotional distress can exacerbate pain and functional limitations, there is a clinical need to clarify how psychological interventions influence these mood states over the course of treatment.

Cognitive Behavioral Therapy (CBT) is an evidence-based psychological treatment for chronic pain [15,16,17]. CBT focuses on pain catastrophizing and pain-related avoidance, which can worsen pain conditions. As psychological therapy is crucial in interdisciplinary pain treatment [18], the effectiveness of Mindfulness-Based Cognitive Therapy (MBCT), a new-generation CBT, is also gaining attention [15].

MBCT is a form of psychotherapy that integrates CBT skills with mindfulness techniques [19]. Mindfulness is the awareness that emerges through paying attention on purpose, in the present moment, and nonjudgmentally to the unfolding of experiences moment by moment [20]. Mindfulness consists of two components: the self-regulation of attention, maintained on present-moment experience, and an attitude of curiosity, openness, and acceptance toward one’s thoughts, feelings, and bodily sensations [21]. Through this nonjudgmental awareness, participants learn to observe their emotional reactions as they arise, which makes mindfulness particularly relevant for monitoring subtle, moment-to-moment mood changes during practice. MBCT may be particularly relevant for patients with chronic pain because mindfulness-based approaches directly target the way patients relate to pain-related thoughts, emotions, and bodily sensations, rather than trying to eliminate these experiences [22]. By fostering non-judgmental awareness and acceptance, MBCT can help patients disengage from maladaptive patterns such as pain catastrophizing and fear-avoidant behaviors, which are key mechanisms maintaining chronic pain and disability [23].

Previous studies have shown that MBCT reduces emotional reactivity to stress and improves affective symptoms such as anxiety and depression in clinical settings [24]. However, these studies only focus on pre- to post-treatment changes, and thus there is a lack of knowledge about the momentary mood states experienced during MBCT sessions. This highlights the need to systematically and quantitatively evaluate both the positive and negative emotional impacts of MBCT.

The impact of individual MBCT sessions on participants’ mood states has not been thoroughly investigated. Patients’ mood states during treatment are an important indicator, as studies in CBT have shown that more positive in-session experiences and deeper emotional involvement are followed by greater symptom improvement by the next session [25]. On this basis, it is plausible that the immediate mood impact of individual mindfulness-based sessions may contribute to subsequent therapeutic change. Although MBCT has been shown to be a safe psychotherapeutic intervention with low invasiveness [26], and has a structured program with predetermined themes and exercises, the specific effects of these themes on mood states remain unclear. For instance, some sessions focus on “directing attention to uncomfortable sensations”; however, the influence of such themes on participants’ emotional states is not understood. By assessing temporary mood states that capture how participants experience each MBCT session, it is possible to identify which sessions contain critical elements that contribute to therapeutic efficacy. Conversely, identifying sessions associated with significant mood deterioration or limited improvement may inform the development of more effective MBCT protocols. If psychologically demanding sessions that have the potential to worsen mood states can be identified, it would be possible for therapists to implement preventive measures accordingly. Thus, clarifying transient mood states during sessions is vital for ensuring the safe and continuous provision of treatment.

Moreover, research on how pre-intervention mood states affect treatment outcomes in MBCT is limited. Previous work in CBT interventions for chronic pain has demonstrated that higher treatment expectations are associated with better clinical outcomes, suggesting that pre-treatment psychological characteristics can influence responses to therapy [27]. Prior meta-analyses of MBCT have shown that higher baseline depression severity is associated with greater benefit from MBCT compared with control conditions, suggesting that initial mood states can moderate the effects of this intervention [28]. Other pre-treatment psychological characteristics, including baseline mood or anxiety levels, may also affect treatment response, but the evidence in chronic pain populations remains limited. Recent work on daily affective dynamics has demonstrated that the intensity and instability of negative affect are robustly associated with higher levels of depressive and generalised anxiety symptoms, and that mood dimensions such as anger-hostility, depression-dejection, tension-anxiety, fatigue, and confusion tend to load on a shared negative affect factor and show strong intercorrelations [29]. Taken together, these results support the hypothesis that baseline mood states, which are themselves interrelated, may jointly influence how patients with chronic pain respond to MBCT. In line with this rationale, the present study adopts an exploratory approach to investigate whether baseline mood states are associated with treatment outcomes. By assuming baseline mood as a potential pre-treatment factor that may shape how participants engage with and benefit from MBCT, we aim to generate preliminary evidence to direct future hypothesis-driven studies on patient selection and the personalisation of mindfulness-based interventions.

This study concentrates on the emotional experiences of patients with chronic pain participating in an MBCT program. First, we aim to characterise the trajectory of multiple mood states (vigor, fatigue, anger-hostility, depression-dejection, tension-anxiety, and confusion) using the Temporary Mood State (TMS [30]) on a session-by-session basis throughout the eight-week intervention. Second, we examine whether baseline mood states are associated with subsequent changes in these mood states over the course of the program in order to explore pre-treatment psychological variables that may influence response to MBCT in this population.

## 2. Materials and Methods

### 2.1. Study Design

This retrospective observational study was carried out using data from an MBCT program delivered as part of routine clinical practice and was not prospectively registered as a clinical trial. The design was chosen to analyze existing clinical data on session-by-session mood states without modifying the treatment protocol or imposing additional assessments on patients, consequently minimizing participant burden while capturing mood changes in a naturalistic setting. This approach enhances real-world representativeness while maintaining ethical feasibility, addressing potential concerns about selection bias. The study protocol was approved by the Institutional Ethics Committee of the Keio University School of Medicine (authorization number 20231048), and all participants provided written informed consent for participation in the MBCT program.

### 2.2. Participants

This retrospective observational study targeted 33 patients who underwent an 8-week MBCT program at the Interdisciplinary Pain Center of Keio University Hospital in Tokyo, Japan, between July 2018 and May 2019.

Patients with chronic pain were referred to the Interdisciplinary Pain Center by physicians from various hospital departments. Those considered suitable for group MBCT by the multidisciplinary team were given written and oral information about the program, and written informed consent was obtained prior to participation. Participants were eligible for MBCT if they (1) were aged 18 years or older, (2) had a diagnosis of chronic pain lasting for more than three months, and (3) were referred to and enrolled in the 8-week MBCT program at the Interdisciplinary Pain Center. Patients were excluded if they had (1) severe cognitive impairment or neurological disorders that would prevent active participation in group sessions, (2) acute psychosis or required priority psychiatric treatment, or (3) insufficient Japanese language proficiency to complete the questionnaires and actively participate in discussions.

### 2.3. MBCT Program

The MBCT program was delivered to patients in its standard format in a group setting, with approximately three to eight participants per group. The program was facilitated by two psychiatrists and one clinical psychologist. The two psychiatrists served as the main facilitators, each with more than 10 years of clinical experience. Both main facilitators had personally completed a standard MBCT program and had been providing individual mindfulness-based psychotherapy for over one year prior to the study. The clinical psychologist acted as a sub-facilitator and supported the delivery of group sessions. To maintain treatment quality and consistency, the therapy team held weekly meetings to exchange feedback and discuss clinical issues arising during the program. The program consisted of eight sessions, with 2 h per session (including a break), once a week at the Interdisciplinary Pain Center. Home practice corresponding to each topic was conducted daily after each session. The program included raisin exercises (eating meditation), body scans, breathing meditation, yoga, walking meditation, and compassion meditation (Table 1).

As a distinctive feature of this study, chronic pain-specified psychoeducation was provided in each session by linking the standard MBCT psychoeducational content. The psychoeducation addressed the biopsychosocial model of chronic pain [31], the role of catastrophizing and rumination [32], and fear-avoidance and pacing [33,34]. Within the biopsychosocial model, particular emphasis was placed on explaining psychological aspects that contribute to the maintenance and exacerbation of pain. Catastrophizing and rumination were introduced alongside exercises that involved noticing thoughts, while psychoeducation about fear-avoidance and pacing was integrated with exercises that focused on recognising automatic pilot mode and habitual patterns of behaviour. Across these components, patients were explicitly encouraged to understand how mindfulness practice could be applied to these pain-related processes, consequently supporting pain reduction and improved coping in daily life.

### 2.4. Measurements

Pain, psychological factors, pain-related disability, and mood states were assessed using self-reported questionnaires. During the first visit, participants completed questionnaires on a tablet device. Subsequently, participants rated their mood states before the start and at the end of each MBCT session.

The primary outcome was the change in TMS mood subscale scores before and after each MBCT session; secondary outcomes included overall changes from the first to the eighth session and associations between baseline clinical variables and mood changes.

#### 2.4.1. Momentary Mood States

The TMS was developed based on the Profile of Mood States (POMS [35]) to assess immediate and momentary mood states. Similar to POMS, the TMS consists of six factors (vigor, depression-dejection, anger-hostility, tension-anxiety, fatigue, and confusion), with items rated on a 5-point scale (from 1 = not applicable at all to 5 = very applicable) and higher scores indicating a better mood. The Japanese version of TMS is reliable and valid (Cronbach’s alpha of the six items ranges from 0.78–0.87 [30]). The TMS is appropriate for detecting short-term mood changes as it evaluates respondents’ current mood state. Therefore, TMS was completed by participants immediately before and after each of the eight MBCT sessions to assess within-session changes in mood.

#### 2.4.2. Pain Intensity

The Numerical Rating Scale (NRS [36]) was used to assess the average pain intensity over the past 24 h using an 11-point scale (from 0 = no pain to 10 = the worst pain imaginable). The NRS has better sensitivity in producing data that can be statistically analyzed compared to other pain-rating scales such as the Visual Analog Scale [37].

#### 2.4.3. Psychological Disorders

The Hospital Anxiety and Depression Scale (HADS) was used to assess anxiety (HADS-A) and depression (HADS-D). This scale identifies anxiety disorders and depression in patients in nonpsychiatric hospitals [38]. The HADS consists of 14 items, 7 referring to anxiety and 7 to depression, which are scored on a 4-point scale (from 1 = very applicable to 4 = not applicable at all). Lower scores indicate a more severe psychological state. Both subscales are valid in the Japanese population (Cronbach’s alpha was 0.81 for HADS-A and 0.76 for HADS-D) [39].

#### 2.4.4. Pain Catastrophizing

The Japanese version of the Pain Catastrophizing Scale (PCS [40,41]) was used to assess pain-related negative thoughts (i.e., magnification: tendency to view the pain as more extraordinary than actual; helplessness: feeling helpless about the pain; and rumination: ruminating on the pain). The score ranges from 0 to 52, with each item rated on a 5-point scale (from 0 = never to 4 = always), and higher scores indicating a greater degree of pain catastrophizing [42]. PCS has demonstrated Cronbach’s alpha coefficients of 0.80 for remuneration, 0.81 for helplessness, and 0.65 for magnification, indicating high internal consistency in Japanese populations [41].

#### 2.4.5. Self-Efficacy

The Pain Self-Efficacy Questionnaire (PSEQ [43]) assesses the degree of confidence in achieving a desired outcome despite pain in daily and social lives. The questionnaire consists of ten items rated on a self-reported scale (from 0 = not confident at all to 6 = completely confident). PSEQ scores range from 0 to 60, with higher scores indicating greater confidence in overcoming pain. The reliability and validity of the PSEQ Japanese version have been established (Cronbach’s alpha = 0.94; intraclass correlation coefficient = 0.80) [44].

#### 2.4.6. Pain-Related Disability

The Pain Disability Assessment Scale (PDAS) was used to assess pain-related disabilities [45]. The scale measures disabilities in daily and social lives in patients with chronic pain and consists of 20 items rated on a self-reported scale (from 0 = pain never interfered with these activities to 3 = pain completely interfered with these activities). Five items refer to activities that strain the lower back, seven refer to activities in daily life, and eight refer to social activities. The scores range from 0 to 60, with higher scores indicating a greater degree of disability. The PDAS has been validated in Japan (Cronbach’s alpha = 0.95 and test–retest reliability = 0.96) [45].

#### 2.4.7. Treatment Expectations

The Credibility/Expectancy Questionnaire (CEQ) is a quick and easy-to-administer scale to measure rational credibility and treatment expectancy [46]. Three items for rational credibility and one for expectancy are rated from 1 (not at all) to 9 (very much); the scores for the other two for treatment expectancy range from 0% (not at all) to 100% (very much). The percentage ratings were subjected to a linear transformation ranging from 1 to 9 to generate a total expectancy score. In total, credibility and expectancy scores ranged from 3 to 27. Higher scores indicate higher credibility and expectancy. Cronbach’s alpha for internal consistency was 0.86 (credibility) and 0.90 (expectancy) [46].

#### 2.4.8. Sleep Disorder

Epidemiological research indicates that both poor sleep quality and insufficient sleep duration can increase the risk of chronic pain [47]. Therefore, sleep disorders were assessed using the Athens Insomnia Scale (AIS), a self-administered psychometric questionnaire. It contains eight items rated on a Likert scale ranging from 0 to 3. Total scores range from 0 to 24, with higher scores suggesting more severe insomnia symptoms. The Japanese version of AIS has been validated (Cronbach’s alpha = 0.88) [48].

#### 2.4.9. Quality of Life

The EuroQol 5-dimension (EQ-5D) is a standardized instrument to assess health status and quality of life (QOL) [49]. It assesses five dimensions (mobility, self-care, usual activities, pain/discomfort, and anxiety/depression) at five levels: no problem, slight problem, moderate problem, severe problem, and unable to do [50]. QOL was calculated from a combination of answers as quality-adjusted life-years. The possible scores range from −0.025 to 1, where 0 represents a health state equivalent to death and 1 indicates perfect health [51]. The Japanese versions of the EQ-5D have demonstrated adequate reliability and construct validity in Japanese populations [52,53].

### 2.5. Statistical Analysis

We used a mixed-effects model to identify the single-session and overall effects of the MBCT program on mood states, adjusted for age, sex, and number of sessions attended. The difference between pre- and post-session TMS scores was defined as the change. An increase in vigor score indicated improvement, whereas a decrease in other TMS scores indicated improvement. Paired *t*-tests were used to identify significant improvements in each TMS score for individual sessions. Cohen’s d was calculated as an effect size (ES). Next, the TMS measures were averaged in both first and eighth sessions, and the difference between these average scores was considered as the overall change. Pearson’s correlation analysis was performed for participants who attended both first and eighth sessions to evaluate the association between overall changes in TMS measures and baseline characteristics, identifying predictors of MBCT program treatment response. *p*-values < 0.05 were considered statistically significant, and Bonferroni correction was applied for multiple tests (statistical significance: *p* < 0.05/8). All statistical analyses were performed using SPSS (version 25.0; IBM Co., Armonk, NY, USA).

No formal a priori sample size calculation was performed since this study included all patients who participated in the MBCT program at the center between July 2018 and May 2019. The analyses should therefore be interpreted as exploratory and hypothesis-generating.

## 3. Results

### 3.1. Baseline Characteristics

**Figure 1:**
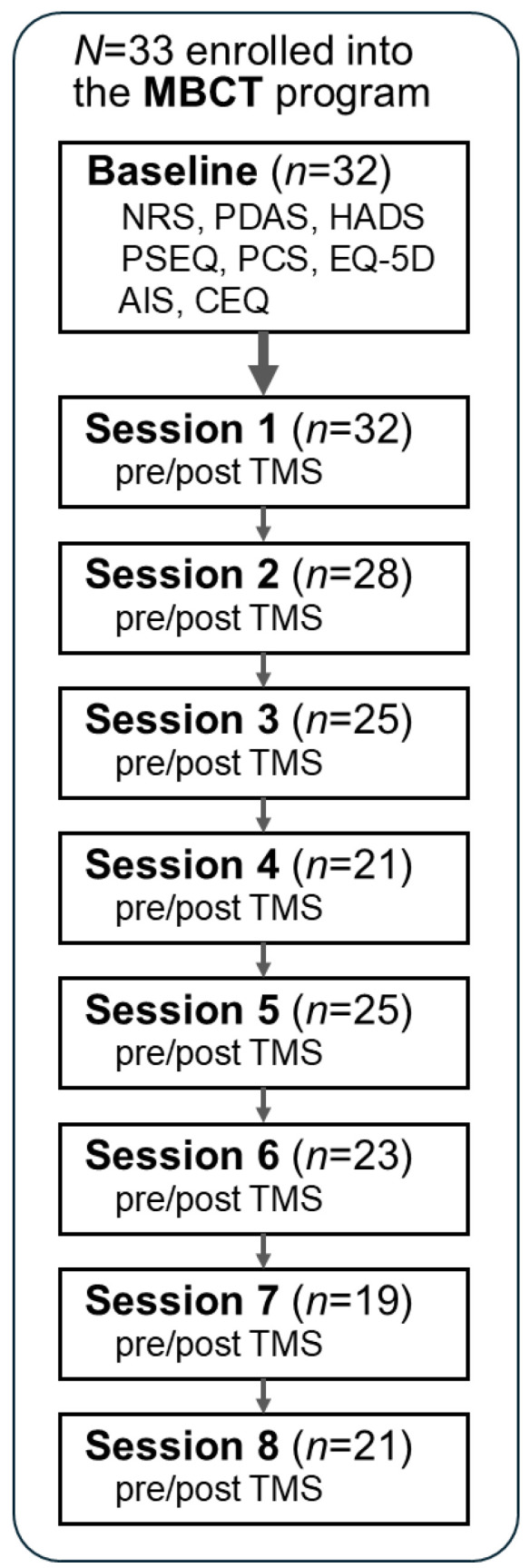
Flow chart of the MBCT program and measures on each session. *N* is the number of participants who attended each session. Abbreviations: MBCT, Mindfulness-Based Cognitive Therapy; NRS, Numerical Rating Scale; PDAS, Pain Disability Assessment Scale; HADS, Hospital Anxiety and Depression Scale; PSEQ, Pain Self-Efficacy Questionnaire; PCS, Pain Catastrophizing Scale; EQ-5D, EuroQol 5-dimension; AIS, Athen’s Insomnia Scale; CEQ: Credibility/Expectancy Questionnaire; TMS, Temporary Mood Scale.

Table 2 presents the baseline characteristics of the participants (*n* = 33). The average age (standard deviation [SD]) was 60.1 years (11.6), and 90.9% of participants were female (*n* = 30). The most common site of pain was lower limb (33%, *n* = 11), and the median duration of pain was five years (quartile range: 2–10). The median number of sessions attended was seven (quartile range: 5–8). The numbers of participants who attended the eight sessions were 32, 28, 25, 21, 25, 23, 19, and 21, respectively (Figure 1).

Baseline pain and psychological measurements were collected from the participants who attended the first session (*n* = 32). The mean pain intensity (SD) before the first session was 5.8 (1.9). Regarding psychological states, the average (SD) HADS-A and HADS-D scores were 7.8 (3.5) and 7.6 (4.5), respectively; the average PCS score was 32.7 (10.6); and the average EQ-5D score was 0.6 (0.1).

### 3.2. The Effect of the MBCT Program on Temporal Mood States

**Figure 2:**
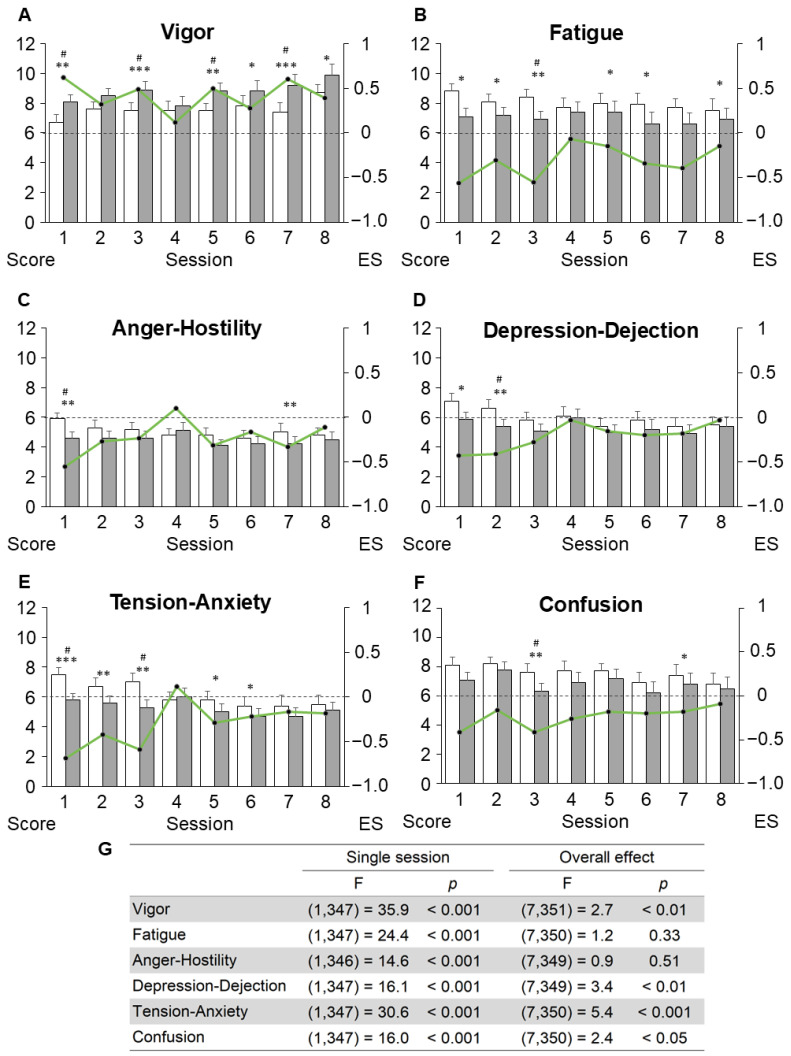
Trajectories of the temporal mood states and effect size in each session. (**A**–**F**) Pre- and post-session scores for the six Temporary Mood Scale (TMS) subscales. Bars represent mean values, and error bars indicate standard errors. Green lines represent the within-session effect size (Cohen’s d) for each session. A paired *t*-test was used to compare the pre- and post-intervention scores in each session. (**G**) Single-session and overall effects estimated using mixed-effect models. ES, effect size. * *p* < 0.05, ** *p* < 0.01, *** *p* < 0.001, # Bonferroni’s corrected *p* < 0.05/8.

The mixed-effects model analysis revealed significant single-session and overall effects on TMS subscale scores (Figure 2).

Significant improvements in vigor (F(7, 351) = 2.7, *p* < 0.01), depression-dejection (F(7, 349) = 3.4, *p* < 0.01), tension-anxiety (F(7, 350) = 5.4, *p* < 0.001), and confusion (F(7, 350) = 2.4, *p* < 0.05) were identified when comparing pre- and post-intervention scores across the entire program.

The paired *t*-test results and ES of the single sessions showed significant improvements in all sessions except the fourth. In session 1 (Noticing automatic reactions), significant improvements were identified in vigor (ES = 0.55, *p* < 0.01), fatigue (ES = −0.55, *p* < 0.05), anger-hostility (ES = −0.60, *p* < 0.01), depression-dejection (ES = −0.45, *p* < 0.01), and tension-anxiety (ES = −0.67, *p* < 0.001). Session 2 (Noticing thoughts and emotions) significantly improved fatigue (ES = −0.35, *p* < 0.05), depression-dejection (ES = −0.45, *p* < 0.01), and tension-anxiety (ES = −0.45, *p* < 0.01). The improved mood states in session 3 (Noticing breath and body) were vigor (ES = 0.61, *p* < 0.001), fatigue (ES = −0.63, *p* < 0.01), tension-anxiety (ES = −0.54, *p* < 0.001), and confusion (ES = −0.63, *p* < 0.01). Session 5 (Accepting) showed significant improvements in vigor (ES = 0.30, *p* < 0.05), fatigue (ES = −0.36, *p* < 0.05), and tension-anxiety (ES = −0.23, *p* < 0.01). In session 6 (Thoughts are not facts), vigor (ES = 0.30, *p* < 0.05), fatigue (ES = −0.36, *p* < 0.05), and tension-anxiety (ES = −0.23, *p* < 0.05) significantly improved. Session 7 (Taking care of yourself) showed significant improvements in vigor (ES = 0.67, *p* < 0.001), anger hostility (ES = −0.40, *p* < 0.05), and confusion (ES = −0.21, *p* < 0.05). Finally, in session 8 (Application for the future), vigor (ES = 0.41, *p* < 0.05) significantly increased. However, no significant change was observed in any mood state in session 4 (Noticing discomfort). Moreover, this session had the lowest mean absolute ES.

### 3.3. Association of the Baseline Characteristics with Overall Change in TMS

**Figure 3:**
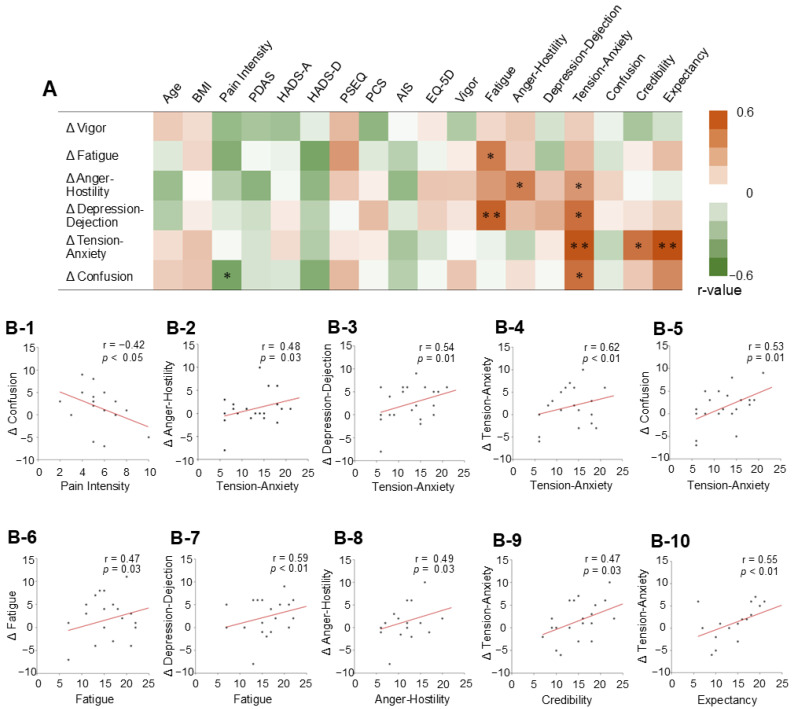
Pearson’s correlation between baseline measures and overall changes in mood state (*n* = 21). (**A**) Heat map showing correlation coefficients (r-values) of the baseline measures and overall changes (Δ) in mood states from sessions 1 to 8. (**B-1**–**B-10**) Scatter plots of the significant correlations. Abbreviations: BMI, body mass index; PDAS, Pain Disability Scale; HADS-A, Hospital Anxiety and Depression Scale-Anxiety; HADS-D, Hospital Anxiety and Depression Scale-Depression; PSEQ, Pain Self-Efficacy Questionnaire; PCS, Pain Catastrophizing Scale; AIS, Athen’s Insomnia Scale; EQ-5D, EuroQol 5-dimension; CEQ, Credibility/Expectancy Questionnaire. * *p* < 0.05, ** *p* < 0.01.

Figure 3 shows the correlation coefficients between the overall changes in TMS measures, baseline pain intensity, and psychological factors. Pain intensity at baseline had a significant negative association with changes in confusion (r = −0.42, *p* < 0.05). Tension-anxiety at baseline was positively associated with changes in anger-hostility (r = 0.48, *p* < 0.05), depression-dejection (r = 0.54, *p* < 0.05), tension-anxiety (r = 0.62, *p* < 0.01), and confusion (r = 0.53, *p* < 0.05). Fatigue score at baseline showed a significant positive association with changes in fatigue (r = 0.47, *p* < 0.05) and depression-dejection (r = 0.59, *p* < 0.01). Anger-hostility at baseline significantly correlated with changes in anger-hostility (r = 0.49, *p* < 0.05). Changes in tension-anxiety scores were significantly positively associated with CEQ credibility (r = 0.47, *p* < 0.05) and CEQ expectancy (r = 0.55, *p* < 0.01).

## 4. Discussion

### 4.1. The Main Strengths of the Study

We conducted a multidimensional evaluation of each session’s and the overall MBCT program’s effect on mood states of patients with chronic pain. Significant improvements were observed in various mood states before and after each session, except session 4. Overall, the MBCT program improved vigor and reduced depression-dejection, tension-anxiety, and confusion. Of note, higher baseline tension-anxiety was associated with larger reductions in anger-hostility, depression-dejection, tension-anxiety, and confusion. This result suggests that participants with higher levels of tension-anxiety at pre-treatment baseline may experience greater improvements in mood states over the course of MBCT.

In this study, the mixed-effects model allowed us to estimate two statistically independent components of mood change: (1) the single-session effect, defined as the immediate pre- to post-session change within each session, and (2) the overall (cumulative) effect, defined as the longitudinal trajectory across the eight MBCT sessions. Thus, the model enabled us to evaluate short-term, session-specific emotional responses and longer-term cumulative improvements independently of one another. However, because MBCT involves progressive skill acquisition and experiential learning, mood changes observed in later sessions may still be influenced by earlier sessions. Therefore, although the model distinguishes these effects statistically, therapeutic processes may still involve inherent carry-over effects. Consequently, the apparent session-specific effects observed here should be interpreted with caution, and designs that explicitly model or manipulate session-to-session dependencies are needed to more precisely isolate the contribution of individual modules. Future studies using designs that explicitly examine or manipulate session-to-session dependencies may help clarify the unique contribution of each session.

The first three sessions showed significant mood state improvements. This positive outcome may be attributed to the effectiveness of psychoeducation, which was initiated at the beginning of the program for mindfulness and painful conditions. The importance of psychoeducation within the MBCT is growing as the revised manual explicitly instructs including psychoeducation for mindfulness in the program structure [54]. In addition, psychoeducation for patients with chronic pain significantly decreased pain intensity and depression [55]. The participants received detailed instructions for managing uncomfortable physical sensations and potentially preventing confusion and feelings of helplessness during the sessions.

The theme in session 4 (Noticing discomfort) minimally improved participants’ mood states. In the MBCT, participants were encouraged to direct their attention to both pleasant and unpleasant sensations that are simultaneously present in their bodies. For beginners, focusing on uncomfortable sensations could trigger negative emotions. In particular, the theme in session 4 was “recognizing aversion,” which involves practicing directing attention toward unpleasant bodily sensations and emotions, which may have heightened psychological burdens and negative mood states. Previous studies have reported side effects of mindfulness meditation on emotional responses, such as anxiety and agitation [56]. However, as the present study program progressed, the participants developed better control over their attention by accepting unpleasant sensations [57]. For patients with chronic pain, accepting bodily sensations of pain without fear or avoidance is crucial in the recovery process to acquire healthy behavioural patterns [58]. Hence, they must acknowledge unpleasant bodily sensations but regulate negative emotions to stay present without being engulfed by them; however, this is challenging. Therefore, detailed and careful guidance may be required to support participants in accepting unpleasant bodily sensations during MBCT for chronic pain. Messages such as “do not try too hard” and “be kind to oneself” were repeatedly conveyed during the sessions.

Of particular note, Session 7 (Taking care of yourself) exhibited significant positive changes that were not observed in other sessions. Specifically, this session achieved improvements in “Anger-Hostility” and “Confusion”—outcomes not attained in previous sessions. This suggests that Session 7 may contain unique therapeutic elements that are distinct from those in other modules. The theme of Session 7 was “Taking care of yourself,” featuring self-compassion exercises focused on self-forgiveness. Interventions that enhance self-compassion have been reported to reduce aggressive tendencies and anger [59]. Importantly, anger is a recognised exacerbating factor in chronic pain [60], implying that anger reduction could potentially improve pain outcomes. Although no overall improvement in “Anger-Hostility” was observed in this study, integrating self-compassion elements into other sessions or homework assignments may enhance the efficacy of future MBCT protocols.

Through the MBCT program, no sessions deteriorated mood states assessed by the TMS. Although many patients with chronic pain are hypersensitive to pain [61] with negative mood states [62], no worsening of mood state was observed from before to after each session. Even though some participants complain about mental symptoms during the MBCT program depending on their conditions [63], addressing pain in the MBCT is unlikely to adversely affect participants’ mood states at a group level.

From a clinical perspective, it is important to identify which sessions within psychotherapy may contain critical elements that most strongly contribute to therapeutic efficacy [64]. Mapping mood changes across sessions can help pinpoint modules that are consistently associated with robust mood improvement and might therefore be prioritised, emphasised, or extended in future adaptations (e.g., sessions focusing on self-compassion or self-care). Conversely, recognising sessions that tend to elicit higher levels of anxiety, confusion, or fatigue (such as those emphasising attention to discomfort) can inform targeted therapist support and risk management strategies during these psychologically demanding components. Previous research on psychotherapy suggests that early recognition and explicit discussion of patients’ distress and negative reactions during treatment can facilitate timely intervention and may reduce the risk of treatment failure or dropout [23]. Within a structured, session-based program such as MBCT, routinely monitoring mood before and after each session offers a practical way to identify particularly beneficial and challenging sessions with the potential to enhance engagement and the overall safety and effectiveness of MBCT for chronic pain.

Mindfulness-based cognitive therapy may improve mood in individuals with chronic pain by targeting cognitive and emotional processes that contribute to the maintenance of negative affect, such as catastrophizing, rumination, and fear-avoidance in response to pain and pain-related cues. Through the cultivation of non-judgmental awareness, decentering from distressing thoughts, and acceptance of unpleasant bodily sensations and emotions, MBCT is believed to enhance emotion regulation and to attenuate the occurrence and severity of negative emotional responses (e.g., fear, anxiety, irritability) triggered by ongoing pain [65]. In the wider literature, mindfulness-based interventions for chronic pain have generally shown small-to-moderate improvements in depressive and anxiety symptoms [66], and the magnitude of change observed in the present study appears broadly consistent with these prior findings, although a direct comparison is limited by differences in design and outcome measures. However, most previous studies examined depression or anxiety using summary indices and did not assess mood states as multidimensional constructs. A distinctive aspect of the present study is the use of a multidimensional mood measure, which enabled us to evaluate several mood dimensions, including tension-anxiety, vigor, anger-hostility, and confusion, and thereby to obtain a more detailed picture of how MBCT may differentially affect particular aspects of mood in patients with chronic pain. Moreover, by focusing on mood states before and after each of the eight MBCT sessions, we were able to explore session-by-session trajectories and to generate preliminary evidence that the thematic content of individual sessions may be associated with distinct patterns of mood change. For example, sessions that emphasise directing attention toward discomfort (session 4) may transiently elicit or maintain negative affect, whereas sessions focusing on self-care along with self-compassion (session 7) may be associated with greater improvements in positive mood. This within-session perspective indicates that the emotional impact of MBCT is not uniform across the program and may help clinicians anticipate when further support is needed and which therapeutic components are likely to be particularly beneficial for improving mood in patients with chronic pain.

The correlation analysis between overall changes in TMS measures and pain and psychological factors at baseline showed that tension-anxiety is most likely the best measure for predicting the overall MBCT effect. Individuals with higher tension-anxiety at baseline exhibited greater responses to the program. Tension-anxiety questionnaire assessed three items (tense, fidgety, and nervous) for both physical and emotional problems. The relaxing effects of mindfulness may have brought physical relief to the participants who were strained. Prolonged emotional strain and stress can lead to a reflexive state of physical tension within the body [67].

Furthermore, chronic stress in muscles maintains a persistent state of guardedness [68]. Although mindfulness meditation was not initially aimed at achieving immediate relaxation [20], a study has observed physical relaxation through meditation [69], suggesting that MBCT may improve physical problems. In addition, the improved emotion regulation skills provided by MBCT may attenuate pre-existing unpleasant emotions. Multidimensional aspects highlight that negative emotions, including anxiety, are often observed in people with chronic pain. The substantial evidence reporting MBCT as an effective treatment for anxiety disorders [70] indicates that it may improve negative psychology in patients with chronic pain. Thus, the physical and emotional aspects assessed by tension-anxiety in TMS are possible targets for MBCT in patients with chronic pain.

### 4.2. Limitations

This study has several limitations. First, the exact effect of MBCT on mood states was unclear because a control group was absent. This retrospective observational study was not designed to test causal efficacy, but rather to characterise within-session and session-by-session mood trajectories under routine clinical conditions. Therefore, the present findings should be regarded as exploratory and hypothesis-generating. Further controlled trials (e.g., with waitlist or active comparators) will be needed to disentangle specific MBCT effects from non-specific or time-related factors, including placebo effects and natural fluctuations over time. Second, the small sample size and bias regarding the participants’ sex (i.e., 90% were women) may limit the generalizability of the findings. To strengthen the reliability and external validity of conclusions about MBCT for chronic pain, future studies should enrol larger, prospectively recruited samples with more balanced sex distributions. Furthermore, the participants had heterogeneous pain characteristics, including etiology and location, and included severe cases that are challenging to treat with standard medical therapy. This clinical heterogeneity reflects the realities of routine tertiary pain care but constrains the precision with which our results can be applied to specific diagnostic subgroups. Again, subsequent research with larger samples should enable stratified or subgroup analyses to clarify whether mood trajectories during MBCT differ according to pain type or clinical phenotype. In addition, the baseline EQ-5D score in this study was lower than the Japanese population norm (0.85), indicating that participants had markedly reduced health-related quality of life at study entry. This suggests that the participants were more severely affected than the general population. Accordingly, the present findings are most applicable to patients with chronic pain and substantial functional limitations, and it remains to be determined whether similar mood patterns would be observed in less severely affected samples. Therefore, caution should be exercised when applying the results of this study to a general population experiencing pain. Further studies with larger and more diverse samples are needed to confirm these results and to clarify the generalisability of MBCT effects in patients with chronic pain. Third, the number of participants varied in each session, and data from the absentees were not collected, suggesting that the intention-to-treat analysis findings may not be accurate. Although mixed-effects modelling can partially accommodate unbalanced data, variability in session attendance and missing observations from absentees may still have introduced bias into the estimated mood trajectories. Prospective designs incorporating strategies to minimise attrition and to systematically document reasons for non-attendance will be important in improving data completeness and interpretability. Fourth, since TMS evaluates immediate and momentary mood states, day-to-day or session-to-session fluctuations may have contributed to variability in the estimated overall effect. While the mixed effects model enabled us to detect cumulative trends across the program, the momentary nature of the TMS may reduce its sensitivity to more stable or persistent mood changes. Consequently, the present findings mainly reflect short-term, within-session mood dynamics rather than enduring changes in affective functioning. To obtain a more comprehensive understanding of MBCT’s impact on mood, subsequent research could integrate such momentary assessments with repeated evaluations of longer-term mood or symptom measures at follow-up to confirm the overall effect.

Additionally, it is possible that not all factors influencing the effectiveness of MBCT were included in the present study. Possible factors include placebo effects and the impact of peer support through interpersonal interactions within the group. In fact, previous research has suggested that positive mood states may lead to higher satisfaction and greater improvement in outcomes [71]. In this study, satisfaction was not assessed; therefore, we were unable to examine the potential long-term prognostic impact of the relationship between mood states and satisfaction. Future studies should conduct more comprehensive evaluations of therapeutic interventions. Including patient satisfaction and perceived helpfulness measures in those studies may clarify whether specific mood trajectories during treatment predict perceived benefit, treatment adherence, and longer-term clinical outcomes.

Moreover, as a retrospective observational study based on existing clinical data, our design is susceptible to selection and information biases and precludes firm causal inferences regarding MBCT’s effects. Prospectively registered, controlled studies with predefined protocols will be essential to validate these preliminary findings and to more rigorously examine causal relationships.

Finally, this study did not consider the group effect of the MBCT program even though participation in a group session is a potential influencer of mood states. Standard MBCT is conducted in group sessions with the primary purpose of practicing listening to and understanding the experiences of other participants without criticism. These approaches may drive the peer support effect in individuals with chronic pain. Future research should consider group effects rather than individual ones. In particular, incorporating measures of group cohesion, perceived peer support, and shared therapeutic factors, and applying multilevel modelling may help disentangle individual-level and group-level contributions to mood trajectories in MBCT.

### 4.3. Clinical Implications and Future Directions

Regardless of the above limitations, the outcomes imply that MBCT may help improve several dimensions of mood in patients with chronic pain and that monitoring session-by-session mood changes can identify sessions that are distinctly valuable or psychologically demanding. Clinically, this information may assist therapists in providing additional support while facing challenging sessions (e.g., those focusing on discomfort) and in emphasising elements such as self-compassion that are associated with greater mood enhancement. Future research with larger, prospectively recruited samples and controlled designs is needed to confirm these outcomes, to examine long-term outcomes, and to further explicate patient-level predictors of response that could inform personalised use of MBCT in chronic pain management. Addressing these limitations through larger, more diverse samples, controlled, prospective designs, explicit modelling of group effects, assessment of patient satisfaction, and longer-term follow-up will be crucial for refining MBCT protocols for chronic pain. Such research may help identify which patients are most likely to benefit and which program components are most critical for optimising mood and functional outcomes.

## 5. Conclusions

In this study, the MBCT program presented improvements in mood states among patients with chronic pain. In addition, those with higher tension and anxiety at baseline exhibited a greater response to the overall effect of MBCT. Except for session 4, the other sessions showed improvements in TMS measures. Given the retrospective design and small sample size, these findings should be interpreted cautiously. Future studies with larger and controlled designs are warranted to confirm the effectiveness of MBCT and further examine baseline psychological factors that may predict treatment response. Although MBCT is an effective treatment option for people with chronic pain, exposure to pain sensations in certain situations may raise concerns about psychological burdens. Because exposure is indispensable during the program, safe and secure approaches and providing an overview of the program, that is, appropriate information in pretreatment guidance, must be considered to maximize the treatment effect.

## Figures and Tables

**Table 1:** Themes and contents of the 8-week MBCT program.

Session	Theme	Education Topics	Exercise
1	Noticing automatic reactions	Learn about mindfulness and autopilot mode	Raisin exercise (eating meditation), body scan
2	Noticing thoughts and emotions	Learn about being and doing modes	Body scan, sitting meditation (focusing on breath)
3	Noticing breath and body	Learn about cognitive-behavioral models	Sitting meditation (expanding focus from breath to body), mindful yoga
4	Noticing discomfort	Learn about pacing	Sitting meditation (noticing reactions to discomfort), 3-min breathing space, walking meditation
5	Accepting	Consider ways to maintain baseline midterm review	Sitting meditation (recalling unpleasant experience)
6	Thoughts are not facts	Learn about cognitive biases, switching thoughts and emotions, and noticing pain precursors	Sitting meditation (observing body, mind, and thoughts), Sitting meditation (find comfort in the body)
7	Taking care of yourself	Create a list of activities that nourish the mind and integrate them into daily life	Sitting meditation (observing body, mind, and thoughts), Sitting meditation (compassion meditation)
8	Application for the future	Review	Body scan

Abbreviation: MBCT, Mindfulness-Based Cognitive Therapy.

**Table 2:** Baseline characteristics.

Sociodemographic Data (*N* = 33)	
Age (years), mean ± SD (min-max)	60.1 ± 11.6 (38–84)
Women, *n* (%)	30 (90.9)
BMI (kg/m^2^), mean ± SD (min-max)	20.7 ± 2.7 (15.9–25.0)
Pain location, *n* (%)	
Lower limb	11 (33.3)
Neck and shoulder	7 (21.2)
Low back	6 (18.2)
Chest	4 (12.1)
Orofacial	3 (9.1)
Other	2 (6.1)
Pain duration (years), median (IQR)	5 (2–10)
Attendance (number of sessions), median (IQR)	7 (5–8)
**Baseline Measurements (*N* = 32)**	**Mean ± SD (min, max)**
Pain intensity	5.8 ± 1.9 (2–10)
PDAS	20.5 ± 9.8 (1–39)
HADS-A	7.8 ± 3.5 (2–16)
HADS-D	7.6 ± 4.5 (0–20)
PSEQ	31.1 ± 14.3 (3–60)
PCS	32.7 ± 10.6 (3–52)
EQ-5D	0.6 ± 0.1 (0.38–1)
AIS	7.4 ± 4.3 (0–19)
TMS	
Vigor	6.5 ± 2.6 (3–15)
Fatigue	8.9 ± 2.9 (3–15)
Anger-hostility	6.0 ± 2.3 (3–10)
Depression-dejection	7.2 ± 2.8 (3–12)
Tension-anxiety	7.6 ± 2.7 (3–15)
Confusion	8.3 ± 2.8 (3–15)
CEQ	
Credibility	15.8 ± 4.9 (7–27)
Expectancy	15.6 ± 5.2 (6–26)

Abbreviations: SD, standard deviation; IQR, interquartile range; BMI, body mass index; PDAS, Pain Disability Assessment Scale; HADS-A, Hospital Anxiety and Depression Scale-Anxiety; HADS-D, Hospital Anxiety and Depression Scale-Depression; PSEQ, Pain Self-Efficacy Questionnaire; PCS, Pain Catastrophizing Scale; EQ-5D, EuroQol 5-dimension; AIS, Athen’s Insomnia Scale; TMS, Temporary Mood Scale; CEQ: Credibility/Expectancy Questionnaire.

## Data Availability

The data are available upon request. The data analyzed in this study are available with the permission of the Institutional Review Board of Keio University School of Medicine based on each request (https://www.ctr.med.keio.ac.jp/rinri/ [accessed on 16 February 2026]).

## Abbreviations

The following abbreviations are used in this manuscript:

MBCT	Mindfulness-Based Cognitive Therapy
TMS	Temporary Mood Scale
CBT	Cognitive Behavioral Therapy
POMS	Profile of Mood States
NRS	Numerical Rating Scale
HADS	Hospital Anxiety and Depression Scale
PCS	Pain Catastrophizing Scale
PSEQ	Pain Self-Efficacy Questionnaire
PDAS	Pain Disability Assessment Scale
CEQ	Credibility/Expectancy Questionnaire
AIS	Athens Insomnia Scale
EQ-5D	EuroQol 5-dimension
QOL	quality of life
ES	effect size
BMI	body mass index
